# Polyamine Oxidation Is Indispensable for Wheat (*Triticum aestivum* L.) Oxidative Response and Necrotic Reactions during Leaf Rust (*Puccinia triticina* Eriks.) Infection

**DOI:** 10.3390/plants10122787

**Published:** 2021-12-16

**Authors:** Marta Dmochowska-Boguta, Yuliya Kloc, Waclaw Orczyk

**Affiliations:** Department of Genetic Engineering, Plant Breeding and Acclimatization Institute—National Research Institute, Radzikow, 05-870 Blonie, Poland; y.kloc@ihar.edu.pl (Y.K.); w.orczyk@ihar.edu.pl (W.O.)

**Keywords:** 1,12-diaminododecane, 2-bromoethylamine, brown rust, diamine oxidase, hydrogen peroxide, hypersensitive reaction, *Lr* gene, polyamine oxidase, resistance, wheat

## Abstract

Hydrogen peroxide is a signal and effector molecule in the plant response to pathogen infection. Wheat resistance to *Puccinia* *triticina* Eriks. is associated with necrosis triggered by oxidative burst. We investigated which enzyme system dominated in host oxidative reaction to *P. triticina* infection. The susceptible Thatcher cultivar and isogenic lines with defined resistance genes were inoculated with *P. triticina* spores. Using diamine oxidase (DAO) and polyamine oxidase (PAO) inhibitors, accumulation of H_2_O_2_ was analyzed in the infection sites. Both enzymes participated in the oxidative burst during compatible and incompatible interactions. Accumulation of H_2_O_2_ in guard cells, i.e., the first phase of the response, depended on DAO and the role of PAO was negligible. During the second phase, the patterns of H_2_O_2_ accumulation in the infection sites were more complex. Accumulation of H_2_O_2_ during compatible interaction (Thatcher and Tc*Lr34* line) moderately depended on DAO and the reaction of Tc*Lr34* was stronger than that of Thatcher. Accumulation of H_2_O_2_ during incompatible interaction of moderately resistant plants (Tc*Lr24*, Tc*Lr25* and Tc*Lr29*) was DAO-dependent in Tc*Lr29*, while the changes in the remaining lines were not statistically significant. A strong oxidative burst in resistant plants (Tc*Lr9*, Tc*Lr19*, Tc*Lr26*) was associated with both enzymes’ activities in Tc*Lr9* and only with DAO in Tc*Lr19* and Tc*Lr26*. The results are discussed in relation to other host oxidative systems, necrosis, and resistance level.

## 1. Introduction

Localized accumulation of reactive oxygen species (ROS) known as the oxidative burst is a plant response to pathogen infection and is an important component of plant resistance. In plants, ROS production was documented for the first time by Doke [1] in potato tubers infected with *Phytophthora infestans*. Results published since then have documented production of different ROS molecules not only in the response to pathogen invasion [2] but also in a number of biological processes including growth, development and reaction to environmental stresses [3]. Reactive oxygen species (ROS) include singlet oxygen (^1^O_2_), hydroxyl radicals (^●^OH), superoxide anion (O^2●−^) and hydrogen peroxide. Production of ROS, which are toxic in higher concentrations, is strictly regulated by ROS-scavenging enzymes. Balance of ROS production and scavenging is maintained by complex red-ox systems [4,5]. The majority of ROS generated in response to pathogen infection is accumulated in the apoplast. Immediately after infection and recognition of the pathogen, the membrane-bound NADPH oxidases and the apoplastic peroxidases are activated and serve as the source of apoplastic ROS in the so-called oxidative response [6].

**Figure 1:**
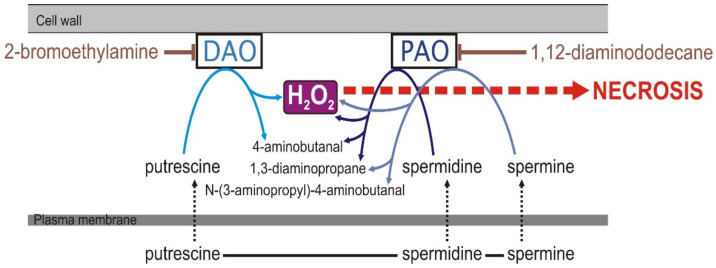
Generation of H_2_O_2_ by DAO and PAO in polyamine catabolism. Metabolic inhibitors of both enzymes and the role of H_2_O_2_ as a signal molecule in Programmed Cell Death and necrosis are indicated. According to modified [8,17].

ROS are key factors in the two-phase oxidative burst and hypersensitive response in plant–pathogen interaction [7]. Hydrogen peroxide with its half-life of about 1 ms is the most stable of all ROS and, therefore, capable of triggering intra- and intercellular signaling and downstream reactions [3]. Apoplastic H_2_O_2_ is generated by diverse enzyme systems: plasma membrane-localized NADPH oxidases (NOX, respiratory burst oxidase homologs, RBOHs), cell wall class III peroxidases (POX), diamine oxidases (DAO) and polyamine oxidases (PAO). The enzymes DAO and PAO generate H_2_O_2_ by oxidation of aliphatic amines, putrescine, spermidine and spermine. Oxidation of putrescine is catalyzed by diamine copper-containing amine oxidase (DAO/CuAO), while oxidation of spermidine and spermine is catalyzed by polyamine oxidase (PAO) [8]. Both enzyme systems form a complex network and are important players in the regulation of cell and tissue differentiation and organ development. They function as regulators of programmed cell death (PCD), which is an indispensable process in both plant differentiation and the immune response [9]. Accumulation of polyamines, elevated expression of PAO- and DAO-encoding genes and increased generation of apoplastic ROS have been reported in diverse plant–pathogen systems: tobacco infected with *Pseudomonas syringae* pv. *tabaci* [10], oat infected with *Puccinia coronata* f. sp. *avenae* [11] and tobacco (*N. tabacum*) infected with a necrotrophic pathogen (*Sclerotinia sclerotiorum*) [12]. All these processes were associated with a hypersensitive response (HR): in barley after powdery mildew infection [13], in tobacco after *Tobacco mosaic virus* (TMV) infection [14] and in tobacco-cultured cells treated with cryptogein, which acted as an HR elicitor [15]. Compatible results of elevated accumulation of putrescine and spermine and higher expression of genes involved in polyamine metabolism were typical not only for host but also for nonhost plant–pathogen systems. The involvement of polyamine accumulation and polyamine oxidation in host HR was found in *Arabidopsis thaliana* infected with *Pseudomonas syringae* and rice infected with *Magnaporthe grisea*. The nonhost HR was reported in tobacco infected with *Pseudomonas cichorii*. Moreover, in this system, silencing of the PAO-encoding gene lowered H_2_O_2_ accumulation and alleviated symptoms of HR. This set of results confirmed the role of PAO in pathogen-induced oxidative burst and pathogen-triggered PCD [16] (Figure 1).

In this study, the wheat cultivar Thatcher and seven near-isogenic lines containing *Lr* resistance genes (*Lr9*, *Lr24*, *Lr25*, *Lr26*, *Lr29* and *Lr34*) were used. The *Lr9* gene, transferred to wheat from *Aegilops umbellulata*, is located on the chromosome 6BL of wheat [18]. This gene confers highly effective resistance and, despite reports about virulent isolates, it is still an important component of wheat resistance to leaf rust [19]. The *Lr19* gene, from *Thinopyrum ponticum*, is located on wheat chromosome 7DL [20]. This translocation also contains the *Sr25* gene, which confers resistance to stem rust [21,22]. The *Lr24* gene transferred from *Thinopyrum ponticum* is associated with *Sr24* stem rust resistance [23]. The *Lr25* gene, from rye *Secale cereale*, located on chromosome 4BS, is associated with the *Pm7* powdery mildew resistance gene [24]. The *Lr26* gene, also from rye, is associated with the *Sr31* gene (resistance to stem rust) and with *Yr9* (yellow rust resistance) [23]. *Lr29* is transferred from *Thinopyrum ponticum* [23]. The *Lr34* gene is responsible for adult plant resistance (APR) against *Puccinia triticina*. It has been used in wheat cultivars for over 50 years and still it is not broken by pathogen evolution [25,26,27,28]. *Lr34* encodes an ABC transporter. The gene has been cloned but the molecular mechanism of the resistance it confers has not been elucidated. It is intriguing that *Lr34* transferred to other, phylogenetically unrelated species still conferred resistance to the pathogens [29,30,31,32].

In this study, we investigated whether the oxidative burst in the wheat–leaf rust system typically associated with necrotic reactions also relies on polyamine oxidation. The results are discussed in relation to necrotic reactions and the resistance level of wheat lines.

## 2. Results

**Figure 2:**
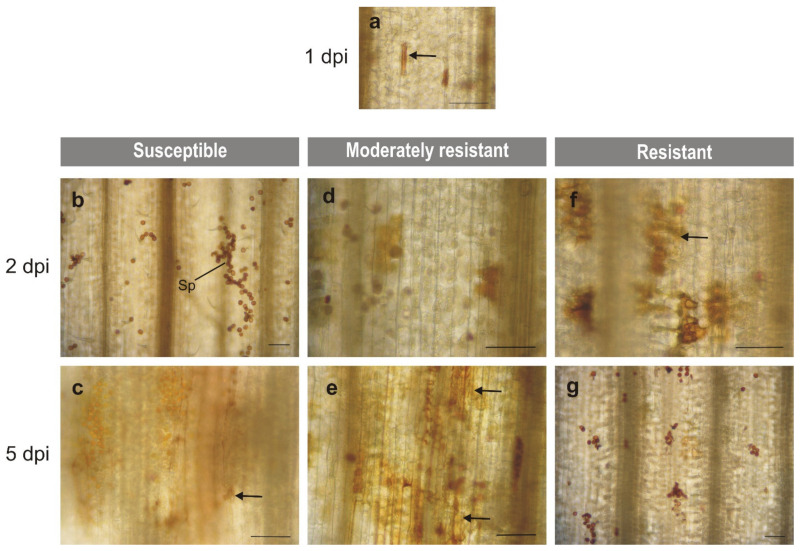
Microscopic localization of hydrogen peroxide accumulation in wheat leaves after inoculation with *P. triticina* spores. (**a**) Representative microscopic image of all tested lines 1dpi; accumulation of H_2_O_2_ was observed in stomata guard cells of all lines. (**b**,**c**) Representative microscopic image of susceptible Thatcher and Tc*Lr34*. (**d**,**e**) Representative microscopic image of moderately resistant: Tc*Lr24*, Tc*Lr25* and Tc*Lr26*. (**f**,**g**) Representative microscopic image of resistant: Tc*Lr9*, Tc*Lr19* and Tc*Lr26*. The arrows indicate H_2_O_2_ accumulation shown with DAB staining. Sp—spore. Bars—100 µm.

The use of DAO and PAO inhibitors allowed the determination of which of these two enzymes played a role in the production of H_2_O_2_ in wheat during infection of *Puccinia triticina*. Strong accumulation of H_2_O_2_ at the infection sites in the susceptible and moderately resistant plants was observed 5 dpi (Figure 2b–e), while strong accumulation of H_2_O_2_ in the resistant plants was 2 dpi (Figure 2f,g).

**Figure 3:**
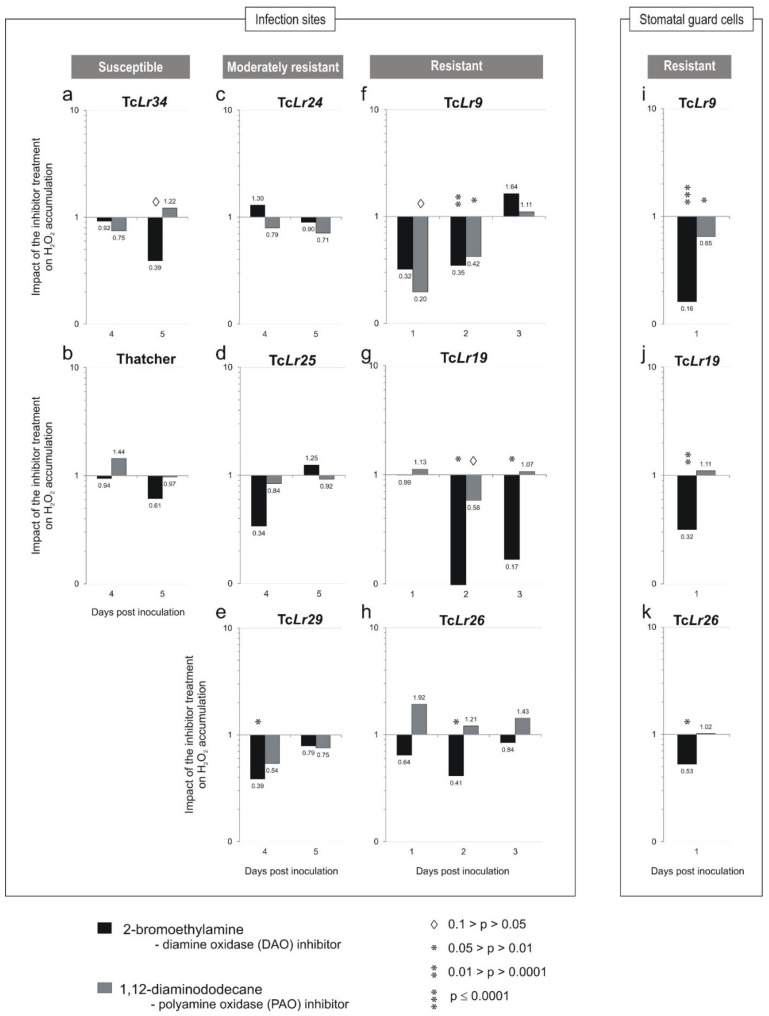
Impact of 2-bromorthylamine, a diamine oxidase (DAO) inhibitor, and 1,12-diaminododecane, a polyamine oxidase (PAO) inhibitor on H_2_O_2_ accumulation in the infection sites (**a**–**h**) and stomatal guard cells (**i**–**k**). Leaves of wheat cultivar Thatcher and isogenic lines Tc*Lr9*, Tc*Lr19*, Tc*Lr24*, Tc*Lr25*, Tc*Lr26*, Tc*Lr29*, and Tc*Lr34* were inoculated with leaf rust spores, infiltrated with inhibitors, and stained with DAB and calcofluor white. The results are presented on a graph with a logarithmic scale, where a value of 1 indicates zero impact (control plants). Values above 1 indicate higher impact of the inhibitor on H_2_O_2_ accumulation, while values below 1 indicate lower impact than in the control. Statistical significance was calculated using the ANOVA test and the LSD post hoc test (STATISTICA 10, StatSoft).

In the susceptible lines (Figure 3a,b), the strongest impact in Tc*Lr34* was 0.39 (5 dpi), while in Thatcher, it was 0.61 (5 dpi). In both cases, the strongest impact was exerted by the DAO inhibitor. In the moderately resistant lines (Figure 3c–e), the strongest impact in Tc*Lr24* was 0.71 (5 dpi) and this was the result of PAO inhibitor treatment, while in Tc*Lr25*, it was 0.34 (4 dpi), the result of the DAO inhibitor, although the changes were not statistically significant. In Tc*Lr29*, the strongest and most statistically significant impact was 0.39 (4 dpi) for the DAO inhibitor.

In the resistant lines (Figure 3f–h), the strongest and most statistically significant impact in Tc*Lr9* was 0.35 (2dpi) for the DAO inhibitor, and 0.42 (2 dpi) for the PAO inhibitor. The strongest and most statistically significant impact in Tc*Lr19* was 0.004 (2 dpi) and, in Tc*Lr26*, 0.41 (2 dpi). In these lines, the impact of the DAO inhibitor dominated; however, in the Tc*Lr9*, the inhibitors of both DAO and PAO enzymes showed a strong impact (Figure 3f).

On the first day after inoculation, H_2_O_2_ was accumulated in stomata guard cells in all tested genotypes, indicating that the response was not specific to one particular type of interaction. The impact of inhibitors was scored in the resistant lines (Figure 3i–k). The strongest and most statistically significant impact on H_2_O_2_ accumulation in stomatal guard cells in Tc*Lr9* was 0.16, in Tc*Lr19*—0.32, and in Tc*Lr26*—0.53. In these lines, the DAO inhibitor effect strongly prevailed.

## 3. Discussion

Accumulation of hydrogen peroxide in the infection site plays an important role in plant defense. In this study, we investigated whether the oxidative burst and necrotic response in wheat infected with leaf rust relied upon the activities of two polyamine oxidation enzymes, and whether these activities depended on the genetic background of wheat lines. The roles of the enzymes were assessed in leaves inoculated with *P. triticina* and infiltrated with the specific inhibitors of the tested enzymes, 2-bromoethylamine (DAO inhibitor) and 1,12-diaminododecane (PAO inhibitor). The results indicated that out of the two tested enzymes, DAO activity prevailed in the oxidative response of wheat to *P. triticina* infection. However, the pattern of this involvement varied in lines with different *Lr* resistance genes.

**Figure 4:**
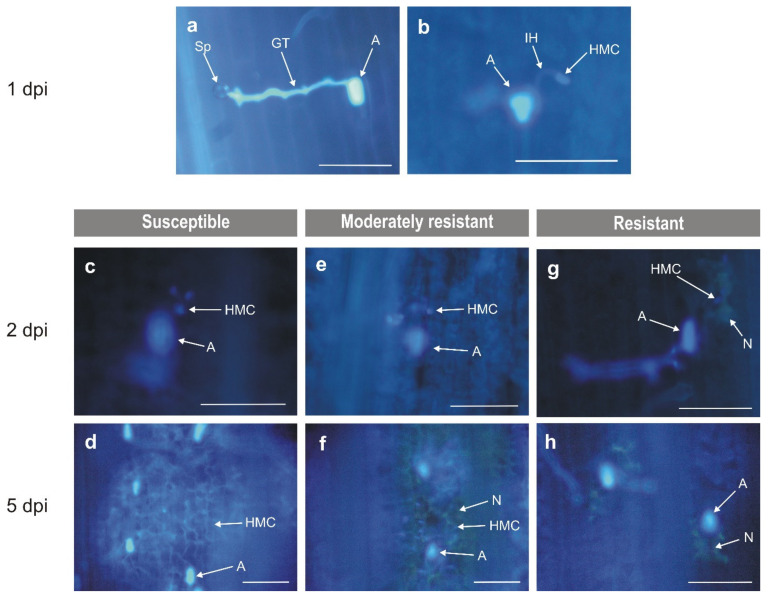
Microscopic observations of plant–pathogen interactions. The observations 1 day post-inoculation were the same for all lines. (**a**,**b**) Representative microscopic image of all tested lines 1dpi. (**c**,**d**) Representative microscopic image of susceptible Thatcher and Tc*Lr34*. (**e**,**f**) Representative microscopic image of moderately resistant Tc*Lr24*, Tc*Lr25* and Tc*Lr26*. (**g**,**h**) Representative microscopic image of resistant Tc*Lr9*, Tc*Lr19* and Tc*Lr26*. Sp—spore; GT—germ tube; A—appressorium; IH—infection hyphae; HMC—haustorium mother cell; N—necrosis. Bars—100 µm.

In susceptible plants (cv. Thatcher, Tc*Lr34*), an oxidative response was observed from 4 to 7 dpi [33]. This coincided with completing the pathogen life cycle and was associated with the development of uredinia, as reported by Orczyk et al. [33]. In these lines, accumulation of H_2_O_2_ was significantly alleviated 5 dpi by 2-bromoethylamine indicating the predominant role of DAO in the oxidative response of susceptible plants. In these plants, NADPH oxidase and class III peroxidases were also involved in the accumulation of H_2_O_2_ [34] (Figure S1 in Supplementary Materials). In moderately resistant lines (Tc*Lr24*, Tc*Lr25* and Tc*Lr29*), accumulation of H_2_O_2_ steadily increased from 1 to 8 dpi [33] and was associated with a necrosis reaction (Figure 4e,f) [33]. However, neither of them was sufficient to fully arrest pathogen growth and uredinia formation. The results indicated participation of both enzymes in the oxidative response; PAO activity prevailed in the response of Tc*Lr24* (Figure 3c), DAO in Tc*Lr25* (Figure 3d) and both enzymes in the response of Tc*Lr29* (Figure 3e); however, only the changes observed in Tc*Lr29* were statistically significant. Additionally, as previously reported by Dmochowska-Boguta et al. [34], the oxidative response in these lines involved type III peroxidases (POX) in Tc*Lr24* and NADPH oxidases (NOX) along with POX in Tc*Lr25* and Tc*Lr29* (Figure S1 in Supplementary Materials).

In resistant lines (Tc*Lr9*, Tc*Lr19* and Tc*Lr26*), the strong oxidative response showed a typical biphasic pattern. The first phase, i.e., accumulation of H_2_O_2_ in stomata guard cells with appressoria (Figure 2a and Figure 4a), was probably induced by pressure generated by appressoria and was observed shortly after inoculation (1 dpi) in susceptible as well as resistant plants [33]. Here, we investigated the effect of the inhibitors on H_2_O_2_ accumulation in guard cells only in resistant plants. The reaction was strongly affected in plants treated with the DAO inhibitor (Figure 3i–k) indicating that, out of the two tested enzymes, the DAO activity prevailed in the generation of H_2_O_2_ in guard cells. Our earlier results showed that NOX and POX [34] (Figure S1 in Supplementary Materials) were involved in this phase of oxidative burst. The present results extend the knowledge and show that the first step of H_2_O_2_ accumulation in guard cells of infected wheat involves DAO and POX activities in all tested lines and, additionally, NOX activity in highly resistant Tc*Lr26* (Figure S1 in Supplementary Materials). The second phase of the oxidative burst is the generation of ROS localized in cells directly affected by germinating spores [33]. In the resistant lines, it was observed as a strong oxidative reaction associated with rapid necrosis (Figure 4g,h). Maximum accumulation of H_2_O_2_ observed 2 dpi was steadily lowered in the subsequent days [33]. The oxidative response in this phase was strongly inhibited by 2-bromoethylamine (DAO inhibitor) 1 and 2 dpi in all tested lines.

Additionally, in Tc*Lr9*, the reaction was also alleviated by 1,12-diaminododecane, indicating the role of PAO activity in this reaction. This set of results complements our earlier reports [34] (Figure S1 in Supplementary Materials) that class III peroxidases and NADPH oxidases were significant sources of ROS in resistant lines after *P. triticina* infection. All four enzyme systems are involved in the oxidative burst in wheat isogenic lines with an *Lr* resistance gene upon inoculation with *P. triticina*. In this study, the inhibitors of the enzymes were used, not a directly measured activity of the enzymes. The former would be important to confirm the role of the enzymes in oxidative burst, necrosis and plant immunity.

Elevated activities of DAO and PAO in response to pathogen infection have been found in diverse plant–pathogen systems. Asthir et al. [35] reported increased activities of both oxidases in wheat inoculated with *Puccinia striiformis* (stripe rust) and the reaction was observed in resistant and susceptible genotypes. A similar oxidative response was noted in barley inoculated with *Blumeria graminis* (powdery mildew). The reaction, which included the accumulation of polyamines and upregulation of DAO oxidases, was observed in both susceptible and resistant plants, but it was stronger in the resistant genotypes [36]. In the same system, the hypersensitive response to powdery mildew was associated with increased levels of free and conjugated forms of polyamines and the elevated activities of both enzymes DAO and PAO. In this system, polyamine accumulation and polyamine oxidation were associated with HR-related resistance [13]. The maize response to infection with the biotrophic fungus *Ustilago maydis* included elevated activity of PAO followed by accumulation of H_2_O_2_ and cell wall lignification [37]. Exogenous spermine, a substrate of PAO, applied to tomato and *Arabidopsis thaliana* induced resistance to the necrotrophic pathogen *Botrytis cinerea*. Combined spermine treatment and pathogen inoculation increased the expression of *PAO* genes, leading to H_2_O_2_ accumulation, a hypersensitive response, and pathogen elimination [38]. Yoda et al. [14] found that polyamine-dependent oxidative burst was a key factor of resistance to *Tobacco mosaic virus* (TMV). They found that the rate of hypersensitive cell death in tobacco leaves infected with TMV was directly associated with synthesis and oxidation of polyamines, and accumulation of H_2_O_2_. Another article from the same laboratory [16] stated that polyamines were the source of H_2_O_2_ in both host and nonhost HRs in higher plants. The authors reported accumulation of polyamines, increased PAO expression, accumulation of H_2_O_2_ and HR reaction in the tobacco response to the non-host pathogen *Pseudomonas cichorii*. Similar reactions in host systems were found in *Arabidopsis thaliana* infected with *Pseudomonas syringae* and rice infected with *Magnaporthe grisea*. As was also reported [39], in the case of *Arabidopsis*, hypersensitive response to the non-host *Pseudomonas syringae* pv. *tomato*, the infection was significantly but not completely blocked by NOX inhibitors, which indicated a role of this oxidative system. Different levels of DAO and PAO activity were observed in barley infected with *Puccinia strifformis* f. sp. *hordei*, depending on the cultivar and isolate [40].

The results presented here show that both DAO and PAO play important roles in the wheat oxidative response to *P. triticina* infection. Participation of the enzymes in the production of hydrogen peroxide was different depending on the *Lr* resistance genes in the wheat line. The general trend showed that the stronger the resistance, the stronger the involvement of PAO and DAO in oxidative burst. Combining the results with our previous findings in the same plant–pathogen system [34] (Figure S1 in Supplementary Materials), we can state that the four systems—POX, NOX, DAO and PAO—participated in the wheat oxidative response to *P. triticina*, although the role of NOX was the smallest. In the susceptible and moderately resistant plants, DAO dominated, but only in Tc*Lr29* and Tc*Lr34* were the changes statistically significant. In resistant plants, an early oxidative response strongly depended on DAO and, in the Tc*Lr9* line, also on PAO.

## 4. Materials and Methods

### 4.1. Plant Materials

Plant material was the wheat cultivar Thatcher and isogenic lines with *Lr* genes Tc*Lr9*, Tc*Lr19*, Tc*Lr24*, Tc*Lr25*, Tc*Lr26*, Tc*Lr29* and Tc*Lr34*, and fungus material was a *Puccinia triticina* isolate. The selected lines were previously characterized by the interaction with the *P. triticina* isolate used [33]. These lines were selected and classified into three groups: susceptible—Thatcher (infection type 4) and Tc*Lr34* (4); moderately resistant—Tc*Lr24* (1), Tc*Lr25* (2) and Tc*Lr29* (1); and resistant—Tc*Lr9* (0), Tc*Lr19* (0;) and Tc*Lr26* (0). Plants were grown in a growth chamber at 22 °C and 16 h photoperiod. Seven-day-old seedlings were inoculated with *P. triticina* spores (0.5 mg/mL water with Tween 20) and placed in the dark at 16 °C and 100% humidity for 24 h. After this time, the plants were transferred back to the growth chamber.

### 4.2. DAO and PAO Inhibitor Treatment and Leaf Staining with Calcofluor White and DAB

Wheat leaves were inoculated with *P. triticina* spores and infiltrated with the inhibitors. The infiltration time was chosen based on our previous results [33]. It was 4 and 5 dpi for the susceptible and the moderately resistant lines [33], and 1, 2 and 3 dpi for the resistant lines [33]. Intact leaves were infiltrated, using sponge corks and clamps, with 2-bromoethylamine (DAO inhibitor) 100 mM in 15 mM NaPi pH 6.5 [41] and 1,12-diaminododecane (PAO inhibitor) at a concentration of 200 µM in the same buffer [42]. The leaves infiltrated with buffers (mock) served as controls. An infiltrated leaf fragment of approximately 2 cm long was marked. After 1 h, leaf samples were cut, collected and stained with 3′3-diaminobenzidine-tetrahydrochloride (DAB) for hydrogen peroxide and calcofluor white to visualize pathogen structures.

Leaf samples were stained in DAB 1 mg/mL pH 3.8 during 4 h in the dark [43,44] and destained in ethanol:chloroform (4:1 *v*/*v*) with trichloroacetic acid 0.15% for 24 h [45]. DAB on contact with hydrogen peroxide turns into a brown precipitate, which was observed under a light microscope.

For calcofluor white staining, the samples were washed twice in ethanol 50%, twice in NaOH 0.05 M, three times in water, once in Tris-HCl 0.1M pH 8.5 and stained in calcofluor white 3.5 mg/mL (Fluorescent brightener 28, Sigma F-6259, Milwaukee, WI, USA) in Tris-HCl 0.1M pH 9.0. The samples were washed once in water and stored in glycerol 25% with 0.1% lactophenol (phenol 1 g/mL:glycerol:lactic acid, 1:1:1, *v*:*v*:*v*) [46]. The infection sites were observed under a fluorescence microscope (Nikon Diaphot, Aizu, Japan, epifluorescence optics with excitation 340–380 nm, barrier filter 420 nm and dichroic mirror 400 nm). The pathogen structures stained with calcofluor white were blue-white. At least 30 infection sites were scored in triplicate biological repetitions per single experimental time-point.

The total numbers of infection sites and DAB-stained (i.e., H_2_O_2_ accumulating) sites were counted, and the percentage of DAB-stained sites was calculated in the inhibitor-treated and in the control plants. The results, shown as the impact of the inhibitor, were calculated according to the following formulas:

$${\%\mathrm{DAB}}_{\mathrm{control}}=\frac{\mathrm{number}\;\mathrm{of}\;\mathrm{infection}\;\mathrm{sites}\;\mathrm{stained}\;\mathrm{with}\;\mathrm{DAB}}{\mathrm{number}\;\mathrm{of}\;\mathrm{all}\;\mathrm{infection}\;\mathrm{sites}}\times 100\%$$

$${\%\mathrm{DAB}}_{\mathrm{inhibitor}}=\frac{\mathrm{number}\;\mathrm{of}\;\mathrm{infection}\;\mathrm{sites}\;\mathrm{stained}\;\mathrm{with}\;\mathrm{DAB}\;\mathrm{after}\;\mathrm{inhibitor}\;\mathrm{treatment}}{\mathrm{number}\;\mathrm{of}\;\mathrm{all}\;\mathrm{infection}\;\mathrm{sites}\;\mathrm{after}\;\mathrm{inhibitor}\;\mathrm{treatment}}\times 100\%$$

$$\mathrm{Impact}\;\mathrm{of}\;\mathrm{the}\;\mathrm{inhibitor}=\frac{{\%\mathrm{DAB}}_{\mathrm{inhibitor}}}{{\%\mathrm{DAB}}_{\mathrm{control}}}$$

The ANOVA test and the least significant difference (LSD) post hoc test (STATISTICA 10, StatSoft) were used for the statistical analysis of %DAB_inhibitor_ and %DAB_control_.

## Data Availability

The data presented in the current study are available from the corresponding authors on reasonable request.

## Supplementary Materials

The following are available online at https://www.mdpi.com/article/10.3390/plants10122787/s1, Figure S1: The impact of NADPH oxidase inhibitor and class III peroxidase inhibitor in Tc*Lr19*, Tc*Lr29* and Tc*Lr34* lines after inoculation with leaf rust spores.
