# Supplementary material for: Polyamine Oxidation Is Indispensable for Wheat (Triticum aestivum L.) Oxidative Response and Necrotic Reactions during Leaf Rust (Puccinia triticina Eriks.) Infection
Source: Plants (Basel). 2021 Dec 16;10(12):2787. doi: 10.3390/plants10122787 (PMC8703351; doi:10.3390/plants10122787)
Supplement: Supplementary file 1 [file plants-10-02787-s001.zip › plants-1511168-supplementary.pdf]

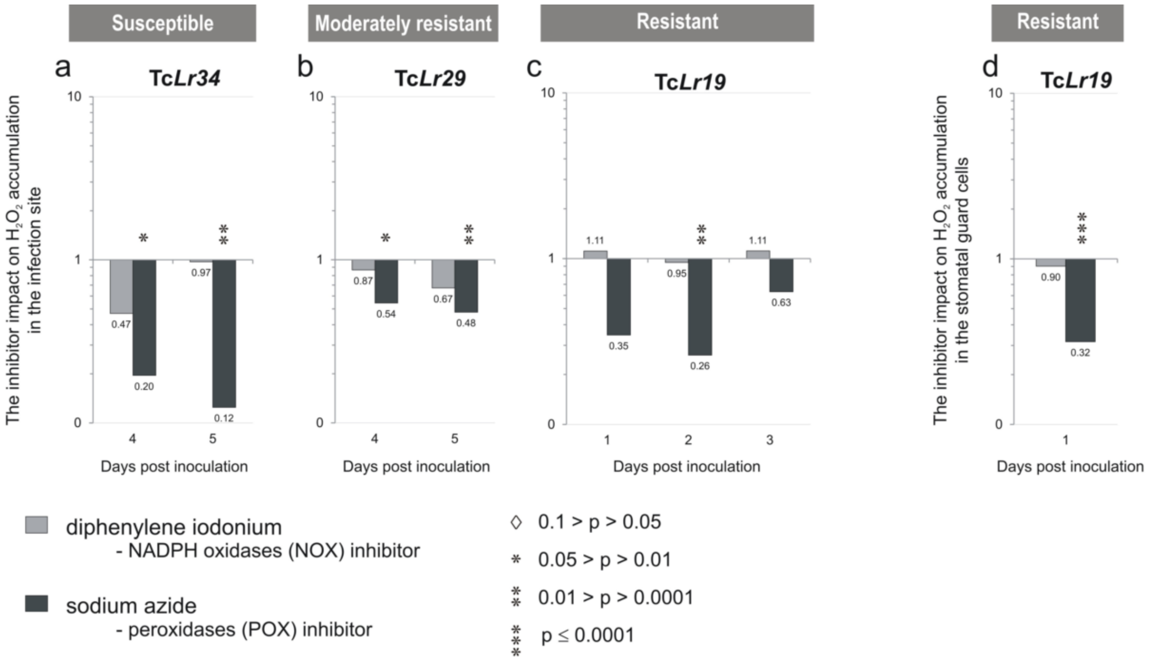

Figure S1. The impact of NADPH oxidase inhibitor and class III peroxidase inhibitor in Tc*Lr19*, Tc*Lr29* and Tc*Lr34* lines after inoculation with leaf rust spores. Leaves were infiltrated diphenylene iodonium (DPI), an NADH oxidase inhibitor (30 μM in 10 mM Tris pH 7.5) and sodium azaide (NaN_3), a peroxidase inhibitor (100 μM in 10 mM Tris pH 7.5) [34] and stained with DAB and calcofluor white, in the infection sites (a–c) and stomatal guard cells (d). The results are presented relative to the control. Statisical significance was calculated using the ANOVA test and the LSD post hoc test (STATISTICA 10, StaSoft).
